# An index to characterize female career promotion in academic medicine

**DOI:** 10.1186/s12995-017-0164-7

**Published:** 2017-07-21

**Authors:** Dörthe Brüggmann, David A. Groneberg

**Affiliations:** Division of Preventive Medicine, Institute of Occupational Medicine, Social Medicine and Environmental Medicine, Goethe-University, Frankfurt, Germany

**Keywords:** Gender, Equity, Index, Career promotion, Academics, Medicine

## Abstract

**Background:**

Imbalances in female career promotion are a key factor of gender disparities at the workplace. They may lead to stress and stress-related diseases including burnout, depression or cardiovascular diseases. Since this problem cannot be generalized and varies between different fields, new approaches are needed to assess and describe the magnitude of the problem in single fields of work.

**Methods:**

To construct a new index, operating figures of female and male medical students were collected for Germany in a period over 15 years and their progression throughout their studies towards specialization and academic chair positions. By the use of different female to male ratios (f:m), we constructed an index that describes the extend by which women can ascent in their academic career by using the field of academic medicine as an example.

**Results:**

A medical student f:m ratio of 1.54 (52,366 female vs. 34,010 male) was found for Germany in 2013. In 1998, this f:m ratio was 0.999. In the same year (2013), the OB/GYN hospital specialists’ f:m ratio was 1.566 (3347 female vs. 2137 male physicians) and 0.577 (516 female vs 894 male physicians) for ENT hospital specialists, respectively. The f:m ratios concerning chairs of OB/GYN and ENT were 0.105 and 0.1, respectively. Then an index was generated that incorporated these operating figures with the student f:m ratio as denominator and the chair f:m ratio as numerator while the hospital specialist f:m ratio served as a corrector in the numerator in order to adjust to the attraction of a given field to female physicians. As a result, the index was 0.044 for OB/GYN and 0.113 for ENT instead of ideally ~1 in a completely gender harmonized situation.

**Conclusion:**

In summary, a new index to describe female career advancement was established for academic medicine. By the use of this index, different academic and medical fields can now be compared to each other and future benchmarks could be proposed. Also, country differences may be examined using the proposed index and the success of specific funding programs.

**Electronic supplementary material:**

The online version of this article (doi:10.1186/s12995-017-0164-7) contains supplementary material, which is available to authorized users.

## Background

In general, occupational health plays an important role in the field of health care personnel [1–8]. Especially working conditions and the question of how to stay healthy are key factors for the well-being of physicians and also their patients [9–13]. Career advancement (also career advancement or professional advancement) is linked to psychological and physical well-being [14]. In this respect, the discrimination of the female workforce by obstacles to promotion [15] may lead or facilitate the occurrence of stress related diseases including burnout or depression or cardiovascular diseases. Since a missing gender equity in medical academics is still present and subject to discussion [16] and with a coming era of too few physicians approaching [17], gender medicine, occupational medicine and related fields need to focus on these issues.

In the media and in scientific discussion, it is not only debated that women earn less payment in equal positions, but also less frequently climb the stairs to high ranked positions, i.e. in the field of medicine [18–23]. However, precise, simple quantifying indices that describe the magnitude of gender imbalance and equity concerning academic career progression, do not exist so far. Therefore, we aimed to characterize this phenomenon in the field of medicine. Our hypothesis was that the permeation of female doctors into highest academic positions does not follow the female to male (f:m) ratios of the medical student population and the physician population. In other words, the pyramid of career success loses the female predominance that is present at the basis, when higher steps are reached. Here, we wanted to construct and establish an index that can be applied when assessing this problem. For this purpose, we used the field of obstetrics and gynecology and compared it to Ear, Nose and Throat (ENT) medicine.

## Methods

### Data

In order to obtain a sound basis of operating figures, we screened the following databases: DeSTATIS database and Federal Chamber of Physicians data base. The DeSTATIS database is an online platform that is maintained by the Federal Statistical Office of the Federal Ministry of Internal Affairs and situated is Wiesbaden, Germany. Amongst numerous federal and country specific data, it publishes numbers of students on a yearly basis. The current set of data encompasses the years 1998, 2003, 2008 and 2013. The database of the Federal Chamber of Physicians, an institution run by the regional chambers of physicians, is also published on a yearly basis and supplies relevant data on the physician demography in Germany.

The online data was retrieved from DeSTATIS files of the Federal Statistical Office at the open access platform [24] and from the Federal Chamber of Physicians at the platform of this institution [25]. As a third set of data that was needed to construct the index, the numbers of full professors/chairs in OB/GYN and ENT were identified by interviews with members of the National Societies of OB/GYN and ENT, internet searches and consultations of journals. In order to determine gender aspects, the proportion of female to male professors was assessed. The numbers of female chairs were exactly given between the period of 1998–2013 in 5 years steps. The exact number of male chairs was only obtainable for 2013. At the other time points there were slight inaccuracies possible, due to difficulties to recall the exact date, when the appointment procedure was finished, the chair finally occupied by a male professor and the sede vacante time was over. Therefore, a number of 36 (assumption that all faculties have a chair) was used and the numbers of female chairs was subtracted.

## Results

### Students

In total, 86,376 medical students were identified in the year 2013 (76,134 Germans). In 2008, there were 79,376 students (Germans: 70,805). In 2003, there were 80,991 students (Germans: 72,013) and in 1998, there were 82,333 medical students (74,139 Germans) (Table 1). Interestingly, the numbers of medical students in Western Germany before the union of 1989 was 85,091 in total, demonstrating that despite the increase of the German population from 63 million Western Germans to 81 million Germans, the number of medical students was not increases, a reason leading to the current situation of a dramatic shortage of physicians.

**Table 1:** Number of medical students in Germany from 1998 to 2013. Retrieved from [24]

Year	Total number medical students			German medical students		
	Total	Male	Female	Total	Male	Female
2013	86,376	34,010	52,366	76,134	29,132	47,002
2008	79,376	30,732	48,644	70,805	26,546	44,259
2003	80,991	34,360	46,631	72,013	30,007	42,006
1998	82,333	41,188	41,145	74,139	36,768	37,371

In the medical student gender analysis of the year 2013, a total of 34,010 male students (29,132 Germans) and 52,366 female students (47,002 Germans) studied medicine in Germany medical schools in 2013. This is a f:m ratio of 1.54 for all female and male students. In 2008, the f:m ratio was 1.583, in 2003 the f:m ratio of 1.357, and in 1998 it was 0.999 (41,145 female vs 41,188 male students) respectively. Altogether, there is steady increase in the percentage of female students over this period of 15 years (Table 1).

#### Specialized physicians and chairs

In 2013, a t total of 17,337 OB/GYN specialists worked in Germany. The f:m ratio was 1.655 for the total (outpatient and hospital) number (10,806 female vs. 6531 male OB/GYN specialists) and 1.566 for hospital specialists. In comparison the f:m ratios were 1.222 for total and 1.178 for hospital specialists in 2008, 0.838 for total and 0.777 for hospital specialists in 2003 and 0.613 for total and 0.548 for hospital specialists in 1998, respectively (Table 2). Generally, the ratio increased towards the present situation with a large majority of female OB/GYN specialists.

**Table 2:** Specialized OB/GYN and ENT physicians and chairs from 1998 to 2013

		OB/GYN			ENT		
		Total specialists	Hospital specialists	Occupied university chairs	Total specialists	Hospital specialists	Occupied university chairs
2013	Total	17,337	5484	42	5952	1410	33
	Female	10,806	3347	4	2051	516	3
	Male	6531	2137	38	3901	894	30
	f:m ratio	1.655	1.566	0.105	0.526	0.577	0.1
2008	Total	16,134	4769	36^a^	5566	1204	36^a^
	Female	8874	2579	2	1762	382	3
	Male	7260	2190	34	3804	822	33
	f:m ratio	1.222	1.178	0.059	0.463	0.465	0.091
2003	Total	15,384	4519	36^a^	5286	1055	36^a^
	Female	7016	1976	2	1519	291	3
	Male	8368	2543	34	3767	764	33
	f:m ratio	0.838	0.777	0.059	0.403	0.381	0.091
1998	Total	14,327	4179	36^a^	5097	956	36^a^
	Female	5447	1479	0	1386	262	1
	Male	8880	2700	36	3711	694	35
	f:m ratio	0.613	0.548	0	0.373	0.378	0.029

^a^The exact number of OB/GYN and ENT female and male chairs was only obtainable for 2013. There are slight aberrations possible in each of the other time points since it was not possible to find exact records, when the ENT and OB/GYN appointment procedures of vacant chairs were completed in each year and chair was finally occupied by a male professor. Therefore, a number of 36 (all faculties had a chair) was used and the numbers of female chairs were subtracted. Physician numbers retrieved from [25]

In the field of ENT, a total of 5952 practicing specialists were identified in 2013 with a f:m ratio of 0.526 for total specialists (2051 female vs. 3901 male ENT specialists) and 0.577 for hospital ENT specialists. In 2008, the f:m ratio was 0.463 for total and 0.465 for hospital specialists, in 2003 it was 0.403 for total and 0.381 for hospital specialists, and in 1998, the f:m ratio was 0.373 for total and 0.378 for hospital specialists, respectively (Table 2). Generally, the increase of the f:m proportion was higher in the field of OB/GYN when compared to ENT indicating in a higher attractivity of OB/GYN for female physicians.

The number of female chair positions was 0 for OB/GYN and 1 for ENT in 1998. It increased towards 4 for OB/GYN and 3 for ENT in 2013 (Table 2).

#### Construction of index

In order to assess the field-specific ascension, the area of OB/GYN was analyzed. The aim was to establish a factor that can be used to assess, describe and quantify if and how women reach top positions in their field, regardless of secondary factors such as culture or parenting. This factor should also be useful for comparison to other fields or countries.

From the different figures that were recorded, the ratio of female to male medical students was chosen as an entry parameter that describes the total of female and male candidates for top positions in OB/GYN. This ratio in 2013 was 1.54. Then, parameters were screened that described job ascension and the f:m ratio of full professor/academic chairs who fulfill in Germany also the task of chief physicians/department heads at the university school of medicine departments, was chosen.

The resulting preliminary index is (1):

1 $$ \frac{f:m\_ ratio\_{chairs}_{field\_ of\_ medicine}}{f:m\_ ratio\_ medical\_{students}_{country}} $$

This index describes the general field specific ascension by which female medical students reach chair positions in a specific field of medicine. This index can be used for different fields of medicine and different countries when data on student and chair numbers are available.

Ideally, this ratio should be 1 in a society that is fully gender-equal.

I.e. OB/GYN in Germany with 36 medical faculties and 36 chairs (2):

2 $$ \frac{f:m\_ ratio\_{chairs}_{OB/GYN}}{f:m\_ ratio\_ medical\_{students}_{Germany}}=\frac{18:18}{1}=1 $$

However, the f:m ratio medical students is not 1 but currently in 2013 (3):

3 $$ f:m\_ ratio\_ medical\_{students}_{Germany}=52366\div 34010=1.54 $$

Therefore, if there would be a gender equality, regardless of bias factors an ideal index with equal distribution of 1 would be (4):

4 $$ \frac{f:m\_ ratio\_{chairs}_{OB/GYN}}{f:m\_ ratio\_ medical\_{students}_{Germany}}=\frac{22:14}{1.54}=1 $$

resulting in 22 female chairs of OB/GYN and 14 male chairs.

When applying this preliminary index to characterize the actual distribution of top academic positions in relation to the starting cohort, the following result is present for OB/GYN for the year 2013 (5):

5 $$ \frac{f:m\_ ratio\_{chairs}_{OB/GYN}}{f:m\_ ratio\_ medical\_{students}_{Germany}}=\frac{4:38}{1.54}=\frac{0.105}{1.54}=0.068 $$

For the field of ENT, it is (6):

6 $$ \frac{f:m\_ ratio\_{chairs}_{ENT}}{f:m\_ ratio\_ medical\_{students}_{Germany}}=\frac{3:30}{1.54}=\frac{0.10}{1.54}=0.065 $$

These results using the preliminary index formulae demonstrate a dramatic difference to the ideal gender equity situation with a value of 1 in both fields on first appearance.

To finalize the index construction and to keep it as simple as possible with only a limited number of operating figures to be drawn from national registers/statistic databases we chose to integrate a factor that mirrors the appeal a given medical field has on female physicians in medical training. Therefore we integrated the f:m ratio of registered physicians of the specific field which can be found in the databases of the federal/national chambers of physicians or ministries of health. This ratio represents a corrector factor for those fields of medicine, which are per se less appealing for women and which therefore do not attract high numbers of female physicians to specify in this field (7):

7 $$ \frac{f:m\_ ratio\_{chairs}_{field\_ of\_ medicine}}{\frac{f:m\_ ratio\_ total\_ specialist\_{physicians}_{field\_ of\_ medicine}}{f:m\_ ratio\_ medical\_{students}_{country}}} $$

For the exemplary field of OB/GYN, our preliminary final index is now for the year 2013 (8):

8 $$ \frac{\frac{f:m\_ ratio\_{chairs}_{OB/GYN}}{f:m\_ ratio\_ total\_ specialist\_{physicians}_{OB/GYN}}}{f:m\_ ratio\_ medical\_{students}_{Germany}}=\frac{\frac{\left(\frac{4}{38}\right)}{\left(\frac{10806}{6531}\right)}}{\left(\frac{52366}{34010}\right)}=\frac{\frac{0.105}{1.655}}{1.54}=0.041 $$

For the exemplary field of ENT, the preliminary final index is now for the year 2013 (9):

9 $$ \frac{\frac{f:m\_ ratio\_{chairs}_{ENT}}{f:m\_ ratio\_ total\_ specialist\_{physicians}_{ENT}}}{f:m\_ ratio\_ medical\_{students}_{Germany}}=\frac{\frac{\left(\frac{3}{30}\right)}{\left(\frac{2051}{3901}\right)}}{\left(\frac{52366}{34010}\right)}=\frac{\frac{0.10}{0.526}}{1.54}=1.23 $$

However, the integration of the general attractivity of a clinical field by using the female to male ratio of total registered specialists in the area may not completely reflect the attractivity towards a career in hospital medicine which ultimately reaches its climax in the position of a clinical chair. In this respect, in the total specialists ratio, there are also the numbers of female physicians included who want to be a specialist in the area but prefer to work in an outpatient setting and not in a hospital setting. This means that they never really aimed for a academic hospital career. To bypass this limit, it is better to integrate the f:m ratio of hospital-based specialists as corrector.

This leads to the final index (10):

10 $$ \frac{\frac{f:m\_ ratio\_{chairs}_{field\_ of\_ medicine}}{f:m\_ ratio\_ hospital\_ specialist\_{physicians}_{field\_ of\_ medicine}}}{f:m\_ ratio\_ medical\_{students}_{country}} $$

For the exemplary field of OB/GYN, our final index is now for the year 2013 (11):

11 $$ \frac{\frac{f:m\_ ratio\_{chairs}_{OB/GYN}}{f:m\_ ratio\_ hospital\_ specialist\_{physicians}_{OB/GYN}}}{f:m\_ ratio\_ medical\_{students}_{Germany}}=\frac{\frac{\left(\frac{4}{38}\right)}{\left(\frac{3347}{2137}\right)}}{\left(\frac{52366}{34010}\right)}=\frac{\frac{0.105}{1.566}}{1.54}=0.044 $$

For the exemplary field of ENT, the final index is now for the year 2013 (12):

12 $$ \frac{\frac{f:m\_ ratio\_{chairs}_{ENT}}{f:m\_ ratio\_ hospital\_ specialist\_{physicians}_{ENT}}}{f:m\_ ratio\_ medical\_{students}_{Germany}}=\frac{\frac{\left(\frac{3}{30}\right)}{\left(\frac{516}{894}\right)}}{\left(\frac{52366}{34010}\right)}=\frac{\frac{0.10}{0.577}}{1.54}=0.113 $$

When comparing the results of the final index of OB/GYN (0.044) and ENT (0.113) to the preliminary construction formulae (formulae 5,6) of OB/GYN (0.068) and ENT (0.065) it becomes clear that the correction factor further optimizes the preliminary index by incorporating the preexisting inclination of the female physicians in training toward a given field of medicine - since it can not be expected that there should be the same number of female chairs when only a lower percentage of female physicians specializes for this field and are able to reach a professorship.

The application of the new index (eq. 10) for the time points 2008, 2003 and 1998 reveals the following changes: Whereas for ENT, the index was 0.077 for 1998, 0.176 for 2003 and 0.124 for 2008, respectively, it was 0 for OB/GYN for 1998, 0.056 for 2003 and 0.015 for 2008, respectively (equations in Additional file 1).

## Discussion

A dramatic shift in the gender proportion of physicians has occurred over the past 25 years and continues to occur as older male physicians retire and a greater proportion of women enter the profession [26]. In this respect, the number of women entering medical schools today exceeds 50%, and the number in hospital specialties is expected to exceed 50% by 2016 [26–28]. As shown here, German numbers of the year 2013 indicate a majority of female medical students (60% of all medical students). As this tendency is also found in the majority of other industrialized countries, numerous previous reports have already discussed the change in gender ratio in the medical profession and coined the expression of a “feminization of medicine”, which also refers to the fact, that the medical profession becomes less dominated by men [26, 29].

However, a precise index to characterize the gender imbalances in academic medicine, does not exist so far. Therefore, we used data from the German OB/GYN field as an example, since this field should per se be a medical field in which women succeed more rapidly, as in other medical fields.

To construct the index, we first concentrated on the denominator and identified the f:m ratio cohort that should represent the baseline. This was not the f:m ratio of the general population or the ratio of the cohort of pupils with high school exams (that allow to study medicine) but the actual number of medical students who study medicine at all German medical colleges at a given time (here the latest available numbers of 2013–12-31 were used). This ratio was 1.54 which points to a vast majority of women that currently study medicine in Germany.

As numerator, a variety of f:m ratios of different operating figures could be used. I.e. ratios of hospital physicians, outpatient physicians etc. However, we wanted to specifically focus on the ability of women to reach the highest positions in an academic field. And this is represented in Germany by full professors/chairs of university departments who traditionally serve as full professors in academic research and teaching and concomitantly as chief department heads in the university hospitals since there is no separation between both parts in Germany for clinical fields of medicine. These positions also form the backbone of the National societies and guarantee the scientific and clinical advancement in their field in the country.

Traditionally, these positions were restricted to male applicants and it took a long time to establish the first “female” chairs. However, regardless of bias factors such as motherhood/parenthood - the overwhelming current dominance of women and already the equal distribution of male and female medical students beginning from 1989 - should have initiated a change in the ratio of chairs in the field of medicine.

By using only an index limited to the medical students gender ratio as basis in the denominator and the chair gender ratio as numerator, the attraction of a specific field of medicine such as OB/GYN to female physicians would not be taken into account. Therefore, we had to integrate an attraction factor and we decided to use the gender ratio of specialized physicians in a single field. These numbers can be found every year in the statistical manual of the Federal Chamber of physicians.

When comparing two fields with an obvious difference concerning attraction to female physicians - OB/GYN with a high percentage of female specialists to ENT with a lower percentage – the calculation of the final index (eqs. 10–12) demonstrates large differences in comparison to the preliminary eqs.. 5, 6. However, the following problem also occurs: When the absolute numbers of chairs are low, the values of the index can be relatively volatile. I.e. In the situation that the numbers of female chairs increase from 1 to 2 out of 36, the index value can double under certain circumstances. Therefore, it is crucial to say that the application of this index does not lead to linear values. Due to the integration of three ratios it has an asymmetrical structure and should be used to analyze trends and directions.

Even with the correction factor for attraction, our new index demonstrates, however, still a very poor picture of gender equity. In striking contrast to the ideal balance of 1 – which is difficult to reach due “biological” bias factors – we here demonstrate that the 2013 index is 0.044 for OB/GYN. Roughly speaking, this is a dramatic underestimation of the female academic work capacity in this field. In an ideal gender-equity situation the indices should have been 1, resulting - in the presence of an attraction bias - in an ideal number of 26 female of 36 OB/GYN chairs instead of 3 current (vs. 10 male instead of 33 current). For ENT, this should be 16 female instead of current 3 female professors.

However, female professors and chairs can not be cloned. Therefore, the index should not be used to propose unrealistic numbers but to define future horizons of improvement for female academics. It should also not be used to allegations to the male chairs. In fact, numerous mentoring programs have been initiated over the past 20 years. Some of them, i.e. the Rahel Hirsch scholarship of the Charité school of Medicine in Berlin, named after the first German (Prussian) women to become a professor of medicine [30], seem to lead to success. However, when reappraising the results of the current index in the fields of OB/GYN and ENT, these programs still seem to be underpowered.

For the field of ENT and the Charité school of Medicine, it can be stated that there is currently no full professor and chair holder present but a vacant academic chair [31]. In this sede vacante situation, three female ENT specialists act as associate professors and acting directors in three separate academic ENT departments at the Charité, but not as full professors and academic chairs since there is no completed election process [31]. It is enticing to speculate if one of them will increase the numbers of female chairs in the nearer future.

One conclusion of the present study is that female mentorship programs need to be enforced. A second and even more important step is that academic funding which is the basis of academic promotion, needs a dramatic shift towards gender-specific programs in order to reform the situation. However, this funding should not be carried out in a scattergun approach but strictly ruled by a direct link of the funding process to a reporting system of the efficacy and benefits.

The former opinion that the female discrimination in academic medicine is strictly related to factors such as “old boys clubs” and networks does not cover the complexity of the problem. In this respect, it can be assumed, that in the year 2015, the majority of German male chairs for OB/GYN do not stick to old overcome pictures of negative role models but try to promote an atmosphere of gender equality in this very specific field of medicine and female health. However, they need the support of specific female academic funding lines and mentorship programs of national funding organizations to boost female progress in medical academics. Our new index indicates that these programs may be too short-tailored for the magnitude of the problem in this field of medicine and science.

When comparing the index between fields of medicine, we used ENT here as an example since the attraction to women is lower. Using the attraction factor, we show that the final index is not that negative as without the factor for ENT. This is in contrast to the OB/GYN results, where even a worsening is seen since the field is very attractive to women. This should also influence the decision making process concerning the establishment of special academic female promotion funding programs in funding agencies and ministries.

What are the reasons for the gender disparity? Both OB/GYN and ENT are surgical fields and it is generally accepted that women are increasingly entering surgical professions [26, 32] although the specialty is still male-dominated, with women representing 10–20% of the surgical workforce according to different studies [26, 28, 33].

Also as shown here and in other studies, the percentage female medical school faculty members holding professor rank remains well below the percentage of men in surgical fields [26, 34]. The reasons are manifold and have been discussed in detail before: family considerations, increased stress and long work hours, sacrifice of personal time, and lack of (or negative) role models are the most common negative factors [35].

A recent meta analysis by Burgos and Josephson stressed that the underrepresentation of women in surgical academia is due to lack of role models and gender awareness [26]. Also, it is not clear whether or not gender itself is a factor that affects the learning of surgical tasks. Unfortunately, this study also pointed to the fact that female students pursuing a surgical career may also experience sexual harassment and gender discrimination that can have an effect on the professional identity formation and specialty choice [26]. The study concluded that bias against women in surgery still exists. There is a lack of studies that investigate the role of women in the teaching of surgery. The study concluded that bias against women in surgery still exists and that there is a lack of studies that investigate the role of women in the teaching of surgery [26]. The fact that women outnumber men in undergraduate enrollments, but they are much less likely than men to major in science or to choose a profession in these fields, was also approached in a recent study by Reuben and colleagues [36]. By using the Implicit Association Test, they showed in an experimental market setting, that implicit stereotypes are responsible for the initial average bias in sex-related beliefs and for a bias in updating expectations when performance information is self-reported [36]. The authors concluded that these stereotypes impair women’s careers in science [36].

## Conclusions

With regard to the presently established index, we have here constructed a relatively simple but robust tool to examine trends in academic gender equity on a yearly basis over different fields of academic medicine and countries. It is of great value to assess if the female dominance in the medical student numbers and physician numbers does also lead to higher chances for women to reach top academic levels. Our index may also be used to analyze if new promotion strategies are successful, i.e. on a 5–10 year periodical basis, since any academic career strategy needs time for resulting changes.

## Additional file

Additional file 1: Equations. (DOCX 525 kb)

## Abbreviations

ENT: Ear, nose and throat medicine
f:m ratio: Female to male ratio
OB/GYN: Obstetrics and gynecology
