# Supplementary material for: An index to characterize female career promotion in academic medicine
Source: J Occup Med Toxicol. 2017 Jul 21;12:18. doi: 10.1186/s12995-017-0164-7 (PMC5521076; doi:10.1186/s12995-017-0164-7)
Supplement: Additional file 1: — Equations. (DOCX 525 kb) [file 12995_2017_164_MOESM1_ESM.docx]

**Equations**

Equation (1)

$$\frac{f:m\_ratio\_chairs_{field\_of\_medicine}}{f:m\_ratio\_medical\_students_{country}}$$

Equation (2)

$$\frac{f:m\_ratio\_chairs_{OB/GYN}}{f:m\_ratio\_medical\_students_{Germany}}=\frac{18:18}{1}=1$$

Equation (3)

$$f:m\_ratio\_medical\_students_{Germany}=52366\div34010=1.54$$

Equation (4)

$$\frac{f:m\_ratio\_chairs_{OB/GYN}}{f:m\_ratio\_medical\_students_{Germany}}=\frac{22:14}{1.54}=1$$

Equation (5)

$$\frac{f:m\_ratio\_chairs_{OB/GYN}}{f:m\_ratio\_medical\_students_{Germany}}=\frac{4:38}{1.54}=\frac{0.105}{1.54}=0.068$$

Equation (6)

$$\frac{f:m\_ratio\_chairs_{ENT}}{f:m\_ratio\_medical\_students_{Germany}}=\frac{3:30}{1.54}=\frac{0.10}{1.54}=0.065$$

Equation (7)

$$\frac{f:m\_ratio\_chairs_{field\_of\_medicine}}{\frac{f:m\_ratio\_total\_specialist\_physicians_{field\_of\_medicine}}{f:m\_ratio\_medical\_students_{country}}}$$

Equation (8)

$$\frac{\frac{f:m\_ratio\_chairs_{OB/GYN}}{f:m\_ratio\_total\_specialist\_physicians_{OB/GYN}}}{f:m\_ratio\_medical\_students_{Germany}}=\frac{\frac{\left(\frac{4}{38}\right)}{\left(\frac{10806}{6531}\right)}}{\left(\frac{52366}{34010}\right)}=\frac{\frac{0.105}{1.655}}{1.54}=0.041$$

Equation (9)

$$\frac{\frac{f:m\_ratio\_chairs_{ENT}}{f:m\_ratio\_total\_specialist\_physicians_{ENT}}}{f:m\_ratio\_medical\_students_{Germany}}=\frac{\frac{\left(\frac{3}{30}\right)}{\left(\frac{2051}{3901}\right)}}{\left(\frac{52366}{34010}\right)}=\frac{\frac{0.10}{0.526}}{1.54}=1.23$$

Equation (10)

$$\frac{\frac{f:m\_ratio\_chairs_{field\_of\_medicine}}{f:m\_ratio\_hospital\_specialist\_physicians_{field\_of\_medicine}}}{f:m\_ratio\_medical\_students_{country}}$$

Equation (11)

$$\frac{\frac{f:m\_ratio\_chairs_{OB/GYN}}{f:m\_ratio\_hospital\_specialist\_physicians_{OB/GYN}}}{f:m\_ratio\_medical\_students_{Germany}}=\frac{\frac{\left(\frac{4}{38}\right)}{\left(\frac{3347}{2137}\right)}}{\left(\frac{52366}{34010}\right)}=\frac{\frac{0.105}{1.566}}{1.54}=0.044$$

Equation (12)

$$\frac{\frac{f:m\_ratio\_chairs_{ENT}}{f:m\_ratio\_hospital\_specialist\_physicians_{ENT}}}{f:m\_ratio\_medical\_students_{Germany}}=\frac{\frac{\left(\frac{3}{30}\right)}{\left(\frac{516}{894}\right)}}{\left(\frac{52366}{34010}\right)}=\frac{\frac{0.10}{0.577}}{1.54}=0.113$$

Equation (13) Final index for OB/GYN in 2008:

$$\frac{\frac{f:m\_ratio\_chairs_{OB/GYN}}{f:m\_ratio\_hospital\_specialist\_physicians_{OB/GYN}}}{f:m\_ratio\_medical\_students_{Germany}}=\frac{\frac{\left(\frac{2}{34}\right)}{\left(\frac{2579}{2190}\right)}}{\left(\frac{48644}{30732}\right)}=\frac{\frac{0.029}{1.222}}{1.583}=0.015$$

Equation (14) Final index for OB/GYN in 2003:

$$\frac{\frac{f:m\_ratio\_chairs_{OB/GYN}}{f:m\_ratio\_hospital\_specialist\_physicians_{OB/GYN}}}{f:m\_ratio\_medical\_students_{Germany}}=\frac{\frac{\left(\frac{2}{34}\right)}{\left(\frac{1976}{2543}\right)}}{\left(\frac{46631}{34360}\right)}=\frac{\frac{0.059}{0.777}}{1.357}=0.056$$

Equation (15) Final index for OB/GYN in 1998:

$$\frac{\frac{f:m\_ratio\_chairs_{OB/GYN}}{f:m\_ratio\_hospital\_specialist\_physicians_{OB/GYN}}}{f:m\_ratio\_medical\_students_{Germany}}=\frac{\frac{\left(\frac{0}{36}\right)}{\left(\frac{1479}{2700}\right)}}{\left(\frac{41145}{41188}\right)}=\frac{\frac{0}{0.548}}{0.999}=0$$

Equation (16) Final index for ENT in 2008:

$$\frac{\frac{f:m\_ratio\_chairs_{ENT}}{f:m\_ratio\_hospital\_specialist\_physicians_{ENT}}}{f:m\_ratio\_medical\_students_{Germany}}=\frac{\frac{\left(\frac{3}{33}\right)}{\left(\frac{382}{822}\right)}}{\left(\frac{48644}{30732}\right)}=\frac{\frac{0.091}{0.465}}{1.583}=0.124$$

Equation (17) Index for ENT in 2003:

$$\frac{\frac{f:m\_ratio\_chairs_{ENT}}{f:m\_ratio\_hospital\_specialist\_physicians_{ENT}}}{f:m\_ratio\_medical\_students_{Germany}}=\frac{\frac{\left(\frac{3}{33}\right)}{\left(\frac{291}{764}\right)}}{\left(\frac{46631}{34360}\right)}=\frac{\frac{0.091}{0.381}}{1.357}=0.176$$

Equation (18) Index for ENT in 1998:

$$\frac{\frac{f:m\_ratio\_chairs_{ENT}}{f:m\_ratio\_hospital\_specialist\_physicians_{ENT}}}{f:m\_ratio\_medical\_students_{Germany}}=\frac{\frac{\left(\frac{1}{35}\right)}{\left(\frac{262}{694}\right)}}{\left(\frac{41145}{41188}\right)}=\frac{\frac{0.029}{0.377}}{0.999}=0.077$$
